# Morpho-Agronomic and Biochemical Characterization of Accessions of Tiger Nut (*Cyperus esculentus*) Grown in the North Temperate Zone of China

**DOI:** 10.3390/plants11070923

**Published:** 2022-03-29

**Authors:** Xiangdong Yang, Lu Niu, Yuanyu Zhang, Wei Ren, Chunming Yang, Jing Yang, Guojie Xing, Xiaofang Zhong, Jun Zhang, Jan Slaski, Jian Zhang

**Affiliations:** 1 Institute of Agricultural Biotechnology, Jilin Academy of Agricultural Sciences, Changchun 130033, China; xdyang020918@cjaas.com (X.Y.); niulu@cjaas.com (L.N.); zyy@cjaas.com (Y.Z.); renwei@cjaas.com (W.R.); cmyang@cjaas.com (C.Y.); jinggyang@cjaas.com (J.Y.); xingguojie@cjaas.com (G.X.); zhongxiaofang@cjaas.com (X.Z.); 2 Faculty of Agronomy, Jilin Agricultural University, Changchun 130118, China; 3 InnoTech Alberta, Vegreville, AB T9C 1N6, Canada; 4 Department of Biology, Okanagan Campus, University of British Columbia, Kelowna, BC V1V 1V7, Canada

**Keywords:** tiger nut, accessions, morpho-agronomic traits, biochemical composition, principal analysis, clustering analysis

## Abstract

Tiger nut (*Cyperus esculentus* L.) has recently attracted increasing interest from scientific and technological communities because of its potential for serving as additional source of food, oil, and feed. The present study reports morphology and biochemical characterization of 42 tiger nut accessions collected from China and other counties performed in the 2020 and 2021 growing seasons at Nongan, Jilin Province. Assessment of variability of 14 agronomic traits including plant height, maturation, leaf width, tilling number, color, size, and shape: 100-tuber weight showed a wide range of phenotypic variation. The color, size, and shape and maturation of the tubers, as well as the leaf width, were the most distinct characteristics describing variation among the accessions. Compositional analyses of major nutritional components of the tubers reveals that this crop could be a source of high-value proteins, fatty acids, and carbohydrates. Specifically, tiger nut tubers contained high levels of starch, oil, and sugars, and significant amounts of fiber, Ca, P, and Na. Furthermore, the tubers appeared to be a good source of proteins as they contain 16 amino acids, including the essential ones. Amino acid profiles were dominated by aspartic acid followed by glutamic acid, leucine, alanine, and arginine. Overall, these results demonstrated that tiger nut is well adapted to the temperature and light conditions in the north temperate zone of China, even with a shorter growth season. The tiger nut accessions collected here exhibited wide variations for agronomical and biochemical traits, suggesting potential for potential for breeding improvement by maximizing the fresh tuber and grass yield based on the optimal selection of genetic characteristics in climate and soil conditions of northern China.

## 1. Introduction

Tiger nut (*Cyperrus esculentus* L.) belongs to a perennial C4 plant of the sedge family (*Cyperaceae*), which produce sweet and nut-like underground tubers with high nutrient value and health benefits [1,2]. Tiger nut originates from the Mediterranean area, where it was domesticated and utilized as one of the important food sources in Ancient Egypt since about 4000 BC [3,4,5,6]. It was reported that tiger nut might be the third oldest domesticated food crop of the ancient Egypt after emmer wheat and barley [7]. During the Middle Ages, tiger nut was introduced into South Europe (i.e., Spain) by Arabic populations after their expansion across North Africa [1]. In 1854, this crop was introduced to the USA and subsequently spread across other American countries, such as Chile and Brazil [2]. Nowadays, tiger nut is widespread in tropical and temperate zones, and exhibits a wide adaptability to diversified environments including natural habitats and fields under cultivation [1,5]. It was reported that tiger nut can even be cultivated in colder climates such as the Netherlands, Switzerland, Germany, Hungary, and Russia [1]. Until now, five botanical varieties of *C. esculentus* (tiger nut) have been identified based on a complete morphometric analysis, including one cultivated variety (*C. esculentus* var. *sativus* Boeckeler) and its four wild varieties (var. *esculentus*, var. *heermannii* (Buckley) Britton, var. *leptostachyus* Boeckeler and var. *macrostachyus* Boeckeler) [5,8,9]. Though being known by the same name and sharing similar morphology, these varieties might have definite geographical origin and distributions, and exhibit a considerable difference in functional applications; one (i.e., cultivated variety) is a commercial crop, while the other four wild relatives are a weed [1,5]. If not specifically designated, the tiger nut in this study means the cultivated type of *C. esculentus*.

Tiger nut can accumulate a great amount of nutrients in its underground tubers, including 25–40% starch, 20–30% oil, 15–20% sugars, and 5–10% protein, and high levels of dietary fibers (8–10%), vitamin C and E (0.08–0.14 g**·**kg^−^1), and minerals, such as calcium, phosphorus, and potassium [1,2,6,10,11]. The tubers are also rich in several bioactive substances such as phytosterols, alkaloids, saponins, tannins, flavonoids, and terpenoids [12,13]. Abundance of nutrients and bioactive compounds in the tubers signifies tiger nut as a valuable crop offering nutritional and health benefits both as human food and animal feed [2,6,13,14]. It was reported that consumption of tiger nut is beneficial to patients with diabetes, cardiovascular disease, and obesity, as it can help boost blood circulation, lessen cardiovascular diseases and thrombosis, prevent stroke and inflammation of the respiratory passages, and reduce the risk of colon cancer [2,13,14,15]. In ancient Egypt, China, and India, tiger nut tubers were also consumed as a liver tonic for healing of stomach pain, mouth and gum ulcers, and as an aphrodisiac for improvement of the human reproductive system [6]. Due to its nutritional properties and health benefits, tiger nut is even dubbed the snack food of the gods, considered as an ideal foodstuff for children, seniors, and the athletes [7]. Tiger nut can be eaten raw, roasted, dried, baked for bread and cakes, or be made into a refreshing beverage [1,2]. In Africa, tiger nut is widely consumed as a sweetmeat or a side dish, whereas in Southern Europe the tubers are mainly used to make a milk-like non-alcoholic beverage called “horchata de chufa”. Tiger nut has become an important crop of the Spanish Mediterranean region with an annual production of 9000 metric tons and importations of another 2300 tons of tubers from African countries [1,2]. Tiger nut is also cultivated in American countries such as Chile, Brazil, and the USA, where it is mainly used as animal feed [2]. 

Several review papers detailing the morphology and development, growth habits, biochemical composition, and cultivation have been recently published [2,6]. Detailed phenotypical and biochemical analyses evaluating tiger nut’s potential and its versatile applications in agricultural and industrial sectors as food, feedstock, and biofuel, and even as a pioneer plant in phytoremediation of polluted areas, have been performed [1,2,6,10,11,14,16,17,18,19,20]. Most research has been focused on nutritional values and health benefits, identification of active ingredients, and development of new products from the tubers for their applications in the agricultural, industrial, and pharmaceutical sectors. Aerial parts of tiger nut, with its long and narrow leaf blades, resemble grass species of the *Poaceae* family. Tiger nut possesses spikelets with distichous glumes and reduced, perianthless flowers, and can produce abundant seeds and tubers facilitating the proliferation of the species [5,21,22]. The underground tubers are initiated and developed at the apex of the rhizome, while the horizontal subterranean stem can produce numerous tillers at the stem nodes, which sprout to generate new seedlings [1,19]. The main storage organs of tiger nut are the underground tubers, with their chemical composition sharing characteristics typical for both the tubers and nuts [2]. Tiger nut is distinctive for its capability of accumulating a significant amount of all three storage reserves including starch, oil, and sugars in the tubers [2,6,11]. Under cultivation, high-yielding tiger nut varieties (about 7.5 tons of tubers per ha, dry weight) can produce approximately 2.25, 1.8, 1.12, and 0.4 tons per hectare of starch, oil, sugars, and protein, respectively. High biomass yields and nutrient component contents make this crop a promising resource of food, oil, and feed production to meet the increasing and diversified demands of the world [6,10]. The unique capability of tiger nut to accumulate a great amount of oil in the tubers provides a novel model system for studying oil accumulation in non-seed tissues [19,23]. Considering its great potential as an important source of food and oil for consumption by humans and animals, tiger nut has attracted increasing interest from the scientific and technological communities. However, tiger nut (cultivated type) is still poorly investigated and underutilized because, in many parts of the world, it is confused with its other four wild relatives that are considered as noxious weeds [1]. 

Tiger nut was introduced into China in the 1960s and 1970s from the late Soviet Union, Bulgaria, and North Korea for cultivation on a small scale on the marginal lands of the country. In recent years, the potential of tiger nut to improve supply of food, oil, and feed for the continuously increasing agricultural and industrial demand has been reconsidered, and increasing cultivation and utilization of tiger nut have been observed in China. In 2019, tuber production reached 100,000 tons from approximately 20,000 cultivated hectares. However, there have been few tiger nut accessions collected and systematically investigated, though their wide distribution across diverse agro-geographical areas of the world. The agronomic and biochemical characteristics of tiger nut accessions are still largely unknown by breeders. Wide collection and characterization of tiger nut accessions are greatly needed for the utilization and genetic improvement of this cultivated crop in the future.

In this study, the morphological and biochemical characterizations were carried out on 42 tiger nut accessions collected in China and other countries between 2014 and 2018. The main objective of the project was to survey the genetic diversity of the agronomic and biochemical traits in this collection to further expand their utilization in the cooler regions of China. 

## 2. Materials and Methods

### 2.1. Plant Materials and Growth Conditions

A total of 42 tiger nut accessions were collected from China and 5 other countries—Ghana, Mali, Cameroon, Spain, and Russia—and maintained in the greenhouse at 26 ± 1.0 °C, 40–60% relative humidity, and a 16 h light/8 h dark cycle. The name, Jilin Yousha Dou, shortened as JYD, were assigned to these 42 accessions. In 2019–2020, field experiments were conducted using a completely randomized block design with three replications on the farm in the Nongan district, Changchun (44.70417° N, 124.92804° E), where tiger nut has been cultivated since 2015. Soil type in this region is classified as sandy texture and the climate is cooler, with an average annual temperature, effective accumulated temperature, and precipitation of 4.7 °C, 2500 °C, and 450–500 mm, respectively. Sulfur-based compound fertilizer (750 kg/ha, N:P:K = 12:18:15) was applied and crops were irrigated 3 times during the growing season. Mature tubers of tiger nut were soaked in water at 30~40 °C for 3 days, and then planted in the soil for continued cultivation in 3-row 6 m × 1.8 m plots. The experimental plots were managed in a similar fashion to that of the local tiger nut crop during the whole growth period. After approximately 120 days after planting, 10 plants from 3 replicates of each accession were harvested and pooled for the biochemical analysis of the storage nutrients in the tubers. 

### 2.2. Morpho-Agronomic Characterization of Tiger Nut Accessions 

Morpho-agronomic traits of selected accessions were characterized and evaluated for their diversity in the northeast China’s climate and growth conditions. The aboveground traits, including plant height and leaf width, were measured 10 days before harvesting. After the harvest, the major traits of the underground organs, including length/width of tubers, tiller number, 100-tuber weight, and the leaf biomass yield, were measured. The morphological traits of individual plants were recorded and expressed as an average of six plants selected at random from the row. Measurements of number of tillers, tuber yield, leaf biomass, and underground tubers were taken from one linear meter selected at random from the row and the final yields were calculated. The abovementioned highly heritable traits were selected for their distinguishability and, hence, they were used for the subsequent analysis.

### 2.3. Analysis of Oil Content in Tiger Nut Tubers

Oil content measurement in tiger nut tubers was carried out as previously described by Turesson et al. [19] with minor modifications. Briefly, a total of 10 frozen tubers from each accession were crushed and ground to a fine powder with mortar and pestle. One gram of each powdered sample was transferred to a 10 mL glass tube containing 4.0 mL methanol: chloroform (2:1) mixture. After centrifugation at 2000× *g* for 5 min, the lower phase containing the lipid extract was transferred to a new tube. Fatty acid methyl esterification was performed using sulfuric acid–methanol (5%, 2 mL), and then quantified by gas chromatography (GC-2010, Shimadzu, Japan)–mass spectrometry (JMSDX-300, JEOL, Japan) to determine the total fatty acid amount, with methyl-heptadecanoate as an internal standard. Oil content of tiger nut tubers was calculated and represented on a percentage dry weight basis. 

### 2.4. Protein Content Analysis

Protein content in tiger nut tubers was determined by the Kjeldahl method according to AACC [24]. The tubers were ground in a mortar, and approximately 2.0 g of the powder was weighed and digested by hot sulfuric acid in the presence of a catalyst (Hg). After titration of the distilled ammonia in the presence of phenolphthalein, 1 mL 0.1 N H_2_SO_4_, bound by the ammonia, corresponded to 0.0014 g nitrogen. The protein content was calculated as the amount of nitrogen multiplied by 6.25.

### 2.5. Analysis of Total Starch, Amylose, and Amylopectin Content

Twenty tubers of tiger nut were dried for 48 h at 70 °C, and then ground to a fine powder in a mortar. Total starch content in tiger nut tubers was measured by the methods of Megazyme Total Starch Assay kit (K-TSTA, Megazyme, Ireland), according to the instruction of the manufacturers. Starch was enzymatically (α-amylase/amyloglucosidase) digested and the hydrolyzed glucose was quantified spectrophotometrically. Amylose and amylopectin content were quantified using the Megazyme Total Starch Assay kit (K-TSTA, Megazyme, Ireland) according to the manufacturer’s protocols. For amylose determination, the samples were defatted using ethanol and were then treated with Concanavalin-A to precipitate and remove the amylopectin. Amylose was enzymatically hydrolyzed to d-glucose by using α-amylase and amyloglucosidase. The total starch, in a separate aliquot, was similarly hydrolyzed to d-glucose. The concentration of amylose was estimated as the ratio of absorbance at 510 nm for the amylose versus that of starch. The amylopectin content of tiger nut tubers was calculated as a difference between the total starch and the amylose content. 

### 2.6. Statistical Analysis

Data collected from 10 agronomic traits and four biochemical characteristics of 42 tiger nut accessions were subjected to statistical analysis. Descriptive statistics, correlation analysis, and one-way variance analysis were performed using SPSS software v.17.0 (SPSS Inc., Chicago, IL, USA). Least significant difference (LSD) was used as a multiple comparison test to express the significant differences between the averages of each accession. Principal component analysis (PCA) analysis was performed using the 14 agronomic and biochemical variables to reduce the complexity of the data. For identification of the comprehensive criteria, the standardized data of each trait were substituted into each principal component, comprehensive value (F-value) was then calculated for each accession and comprehensive criteria was selected for assessing tiger nut accessions.

## 3. Results

### 3.1. Variations of Agronomic and Biochemical Characteristics among 42 Tiger Nut Accessions in Northern Regions of China

A total of 42 tiger nut accessions collected from China and five other countries were characterized based on field observations in Jilin, China. The accessions exhibited a similarity in morphology for their aboveground parts, though some differences in the leaf shape (plant height, leaf width, and length/width ratios) were observed among the accessions. However, contrary to the previous works reporting that tiger nut can produce a copious number of seeds and thus propagates by both the seeds and the underground tubers [5,21,22], all but two of accessions from Cameroon (JYD-35 and JYD-36) examined in this study showed no flower structure under field conditions, and no viable seeds were produced. For the underground growth, large differences were observed for tuber characteristics such as shape, color, and size among the accessions (Table 1). Accession JYD-41 from Russia had the largest size (25.3 ± 2.76 cm) of tuber and accession JYD-35 from Cameroon exhibited the smallest size (9.52 ± 1.64 cm) of tuber. Based on the length/width ratio of the fresh tubers, a total of 12, 18, and 12 accessions with round, oval, and long tubers, respectively, were identified. Furthermore, most of the accession produced yellow tubers, and only one accession from Cameroon (JYD-34) had red tubers (Table 1). Since the tubers are the predominantly utilized part of crop, their morphological characters (shape, color, and size) are typically employed to classify the accessions [11,25]. 

Ten major agronomic traits and four biochemical characteristics were further examined under field conditions. The basic descriptive statistics, such as the minimum, maximum, averages, and coefficient of variation (CV), for each character, are provided in Table 2. Considerable variations among the accessions with regard to the 14 characteristics were observed. Analysis of one-way variance showed significant differences (*p* < 0.01) in five aboveground traits, such as fresh tubers, tiller number per plant, 100-tuber weight, and fresh weight of leaf. However, no significant differences were found in plant height, suggesting the minimum contribution of this character to the variability among the tested accessions (Table 2). Higher variations (21.81–50.83% of CV) were found in the agronomic traits such as length/width ratio of fresh tubers, tiller number per plant, 100-tuber weight, and fresh weight of aboveground leaf and underground tubers, while four biochemical components—starch, oil, sugars, and protein contents—exhibited a lower variability (8.96–10.80% of CV). Other traits (width and length/width ratio of lead, length, and width of tuber) showed a moderate variation (15.63–19.10% of CV). The largest variations were observed for two yield-related traits—tiller number (50.83% of CV) and 100-tuber weight (30.37% of CV). The identical ratings were attained for both the underground growth and aboveground agronomic traits in the supplementary field experiments conducted in 2020 and 2021. This observation implies that environmental factors may exhibit low effect on the reported variability among the accessions (Table 2). 

To evaluate variations of agronomic and biochemical traits, the accessions were assigned to 3 groups based on the tuber shape, including 28 accessions with an oval tuber, 12 accessions with a round tuber, and 12 accessions with a long tuber (Table 1). Remarkable differences among the tiger nut accessions of different shape of tubers were identified (Table 3). Generally, the accessions with long tubers (group III) had narrower leaves and tubers of smaller diameter and higher length/width ratio than those classified as round (Group I) or oval tubers (Group II). The leaf characters correlated with the shape of the tubers. Specifically, the group III had more (~2-fold) tillers than the other two groups, while its 100-tuber weight showed no significant difference as compared to the other groups. Moreover, the yield of both leaves and tubers was the highest in the group III, suggesting that the tiller number is a primary factor driving the yield of below- and aboveground biomass. As for the biochemical traits, the group III comprises of accessions with higher protein content than group I and group II, while there was no significant difference in starch, oil, or sugars content among the three groups. Thus, the shape of tiger nut tubers might provide practical information to be used in the breeding and selection of this crop. 

### 3.2. Correlation Analysis of 42 Tiger Nut Accessions

Correlation analysis was performed to further analyze the relationships among the agronomic and biochemical traits under field conditions of the cooler region of China (Table 4). Leaf length/width ratio was positively correlated with the plant height and tuber length/width ratio, but negatively correlated with the width of leaf and the tuber, suggesting that the tiger nut accessions with long tubers typically have a higher tuber length/width ratio. Tuber length/width ratio was positively correlated with tuber length and leaf length/width ratio, while negatively correlated with the width of leaf and tuber. The 100-tuber weight was positively correlated with the tuber length, length/width ratio of leaf and tuber, and tiller number. The number of tillers was positively correlated with tuber length and the length/width ratio, while being negatively correlated with the tuber width. Tuber yield was positively correlated with the tiller number, tuber length, and length/width ratio, while showing no significant correlation with 100-tuber weight, suggesting that the high-yielding tiger nut accessions tend to have a higher tillers number, longer tubers, and high length/width ratio. On the other hand, leaf yield was significantly correlated with tiller number, tuber length, and length/width ratio of leaf and tuber. 

With respect to the biochemical traits, the protein content was negatively correlated with oil and starch, but not correlated with the sugar content. Oil and starch content in the tubers was positively correlated, and starch and sugar content were negatively correlated (Table 4). Interesting associations between the agronomic and biochemical components among the tested accessions were also found. For instance, the protein content was positively correlated with the number of tillers and the tuber length/width ratio, but negatively correlated with the plant height, width of leaf, and diameter of the tuber. Oil content was positively correlated with the plant height and tuber width, while negatively correlated with the number tillers and tuber the length/width ratio. Starch content was positively correlated with 100-tuber weight, while sugars content was negatively correlated with 100-tuber weight (Table 4). 

### 3.3. Principal Component Analysis of the 42 Accessions

The principal component analysis (PCA) taking into consideration 14 agronomic and biochemical variables was performed to further explore traits’ contribution to the observed variability among the accessions. The results revealed that the first four principal components had Eigen values greater than one and cumulatively accounted for 74.07% of the total variations among the accessions (Table 5). The first component alone explained 35.18% of the total variation and was mainly associated with the tuber length/width ratio, tiller number and tuber width. The second principal component explained 17.01% of the variation and was predominantly associated with 100-tuber weight and the tuber length. The third principal component explained 12.6% of the variation and was mainly associated with the leaf length/width ratio and the plant height. The fourth principal component accounted for 9.27 % of the variation, and was largely associated with the sugar and starch content (Table 5). These results provide helpful insight to the process of selection and breeding new tiger nut cultivars. 

### 3.4. Comprehensive Assessment of 42 Tiger Nut Accessions

For the comprehensive assessment of these 42 tiger nut accessions, the standardized data of each trait were substituted into each principal component, and comprehensive values (F-value) was calculated for each accession. The results showed that four accessions (JYD-27, JYD-41, JYD-20, and JYD-29) had higher F-values, suggesting that these accessions with long tubers exhibited better comprehensive characters compared with the other accessions (Table 6). Correlation analysis indicated that the F values were positively correlated with the tiller number, tuber length, tuber length/width ratio, yield of tuber and grass, and protein content, while being negatively correlated with the tuber width and oil content (Table 7).

### 3.5. Clustering Analysis of the Tiger Nut Accessions

**Figure 1:**
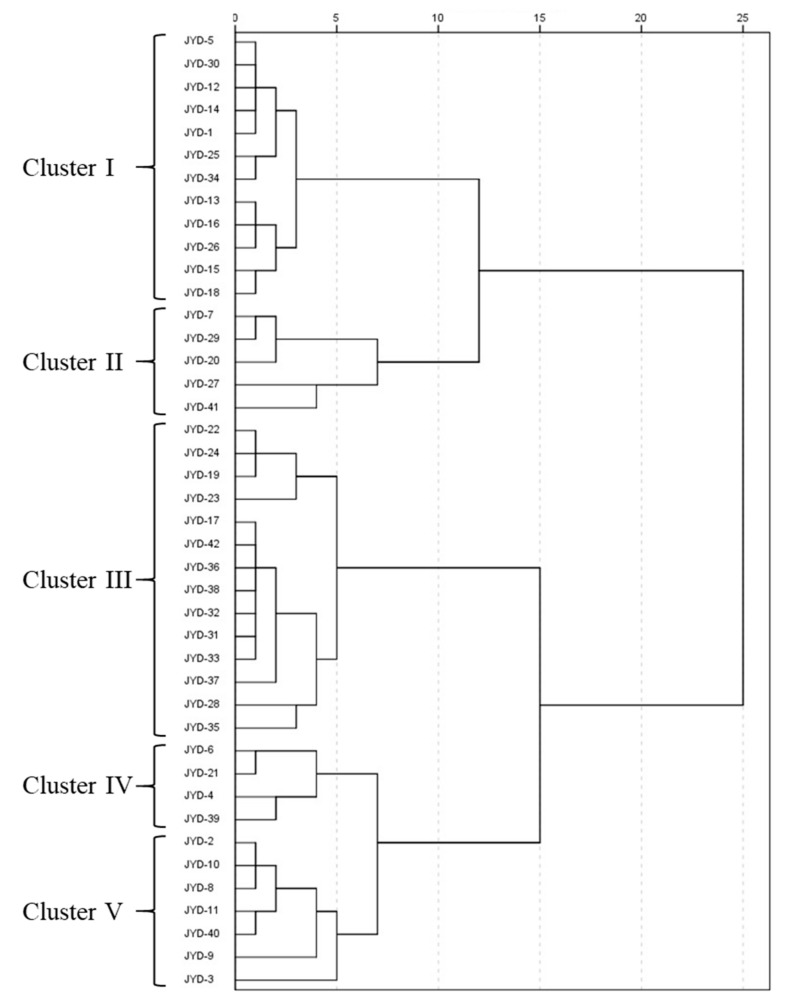
Cluster analysis of 42 tiger nut accessions based on the 14 agronomic and biochemical traits.

Cluster analysis that was performed based on the variability of 10 agronomic and four biochemical traits among these accessions generated five major clusters with the distance between clusters ranging from 0.0 to 2.5 (Figure 1). The number of accessions belonging to each cluster varied from 4 in clusters IV to 14 in cluster III. Cluster I included 12 accessions. Cluster II contained 5 accessions (JYD-7, JYD-20, JYD-27, JYD-29, and JYD-41) which showed higher tiller number, tuber length/width ratio, yield of tuber and leaf, protein content, and lower tuber width and oil content. Cluster III, Cluster IV, and Cluster V comprised of 14, 4, and 8 accessions, respectively. The cluster analysis involving the agronomic and biochemical traits revealed that tiger nut accessions collected from 35 locations across China showed no definite distributions and they were dispersed across the 5 clusters. Three accessions from Cameroon were dispersed in Cluster I and Cluster III, and three accessions from Ghana, Mali, and Spain were included in Cluster III. The Russian accession JYD-41 was contained in Cluster II. These results should be considered a valuable resource for the future breeding programs since they could facilitate identification of the optimal parental combinations offering considerable phenotypic diversity of the candidate accessions of different geographical origin. 

## 4. Discussion

As a sub-cosmopolitan plant, tiger nut is widely distributed in the tropical and temperate regions of the world [5]. Wide adaptability to many adverse habitats allows this crop to extend to other areas, such as marginal or desert fringe lands [16]. Cultivation of tiger nut generally requires mild climate above 30 °C and sandy soil [1]. The crop can propagate by both the seeds and underground tubers [1,5]. In this study, adaptability of these 42 tiger nut accessions was surveyed based on field observation of trials established in the northeast regions of China. Tiger nut tubers showed no dormancy and sprouted when the soil temperature stays above 12 °C, though the sprouting rate might be improved with higher temperatures [1]. After the emergence, the plants grow rapidly and can cover the land in about two months. Formation of the tubers begins 40–50 days after emergence. In this region, cultivation cycle of *C. esculentus* is approximately 120–130 days. In general, our observations suggest that tiger nut is relatively well adapted to the agricultural production conditions in the cooler regions of China and despite the shorter growing season it can successfully develop the tubers accumulating beneficial nutrients. However, no flowering (except for JYD-35 and JYD-36) and seed-setting were observed among the 42 examined tiger nut accessions, which were able to propagate by the underground tubers. This finding remains in disagreement with some previous studies reporting capability of profuse seed production [5,21,22]. The reason for detected discrepancy might be due to the differences in the photoperiod and/or temperature between cooler regions of China and the typically warmer regions of crop domestication. Our primary studies showed that tiger nut possesses numerous and small chromosomes (3n = 156) (data not published). Odd number of chromosomes might cause lead to agmatoploidy or qualitative aneuploid chromosome number change, thus resulting in abnormal flowering and seed setting. The specific mechanism impacting the generative phase needs to be further addressed. 

Extensive collection and characterization of the accessions is essential for the improvement programs of tiger nut. To date, scarce reports focusing on the comprehensive characterization of agronomic and biochemical traits and genetic diversity analysis of the tiger nut accessions have been published. The classification of tiger nut was generally performed based on the tuber characteristics such as shape, color, and size [1,11]. In the present study, 42 tiger nut accessions collected from China and several other countries were characterized for 10 agronomic and 4 biochemical traits under field conditions. Our results indicated that wide variations were observed among the accessions. Among them, most of the agronomic traits exhibited higher variations than the biochemical traits. The largest variations were observed for two yield-related traits—the number of tillers per plant and the 100-tuber weight—suggesting that these two traits should be considered in the breeding program aimed at increasing tuber yield of tiger nut. Clustering analysis showed that all 35 accessions collected in China were dispersed across 5 clusters. This observation may suggest that the tiger nut accessions currently cultivated in China were introduced in the last century from the same or similar in terms of climatic conditions jurisdictions. Establishing the provenance of the past introductions would be difficult to determine at the moment. 

To encourage the use of the tested set of tiger nut accessions in breeding programs focused on the development of new varieties adapted to the conditions of northern China, we carried out correlation analysis on 14 traits. The results revealed wide correlation among the above- and underground traits. The leaf length/width ratio showed significant correlation with that of the tubers. The number of tillers was positively correlated with the tuber length and the length/width ratio. Both tuber and leaf yield were significantly correlated with the tuber number, tuber length, and length/width ratio. These results imply that the tuber number and length/width ratio could be good reference characters to maximize fresh tuber or aboveground biomass yield. Accessions with the higher tuber number and length/width ratio commonly displayed higher tuber or leaf yield, and higher protein content. 

Based on the results of the principal component analysis, 4 components were selected, which accounted for 74.07% of the total variations among 42 tiger nut accessions. Tuber traits, such as the tuber length/width ratio, the tiller number, and 100-tuber weight, showed greater contribution in the first and second principal components. Thus, selection of accessions based on these traits would be most effective tool for comprehensive assessment of the germplasm and would facilitate detection of the most promising candidates for the future improvement programs of tiger nut. The results showed that three accessions from China (JYD-27, JYD-20, and JYD-29), and one accession from Russia (JYD-41) possess the highest compounded value among all tested accessions since they feature long tubers and high tiller number and offer the highest yields of tuber and aboveground biomass. 

In the present study, four major biochemical components (starch, oil, protein, and sugars) were investigated. Since previous studies revealed the differences in specific composition of the biochemical traits of the tiger nut tubers [11], further detailed analyses of protein and oil in the tubers from the Jilin collection are warranted.

## 5. Conclusions

The present study examined a battery of the agronomic and biochemical characteristics of 42 tiger nut accessions grown under field conditions of the cooler regions of northeastern parts of China. The collected data revealed existence of genetic variability among the cultivated tiger nut genotypes from China and several other tiger-nut-growing jurisdictions. While tiger nut appears to be reasonably well adapted to the cooler agro-climatic conditions, the tested accessions were unable to produce viable seeds, and only few of them managed to develop non-fertile flowers. The highest variability was found with respect to several agronomic traits, including the number of tillers per plant, the 100-tuber weight, the length/width ratio of fresh tubers, and the fresh weight of leaf and tubers. On the other hand, the biochemical components, such as starch, oil, sugars, and protein, exhibited fairly low variability. Different degrees of correlation were noted among the tested agronomical and biochemical traits of this collection, with the first principal component accounting for 74.07% of the total variations. The compounded crop value was positively correlated with the number of tillers, tuber length, tuber length/width ratio, yield of tuber, and leaves and protein content, while it was negatively correlated with the tuber width and the oil content. 

## Figures and Tables

**Table 1:** Tuber traits of tiger nut accessions used in the study.

Accessions	Shape	Color	Length (cm)	TLWR	Origin
JYD-1	Long	Yellow	18.65 ± 1.2	2.11	Hubei, China
JYD-2	Oval	Yellow	15.42 ± 1.4	1.36	Hubei, China
JYD-3	Long	Yellow	17.77 ± 1.84	1.84	Hubei, China
JYD-4	Oval	Yellow	15.3 ± 0.82	1.25	Hubei, China
JYD-5	Long	Yellow	16.91 ± 0.94	1.86	Hubei, China
JYD-6	Round	Yellow	14.26 ± 0.9	1.15	Hubei, China
JYD-7	Long	Yellow	17.55 ± 1.34	2.18	Hubei, China
JYD-8	Oval	Yellow	14.9 ± 0.84	1.25	Hubei, China
JYD-9	Oval	Yellow	15.37 ± 0.96	1.23	Hubei, China
JYD-10	Oval	Yellow	14.32 ± 1.55	1.28	Hubei, China
JYD-11	Round	Yellow	14.24 ± 0.8	1.19	Hubei, China
JYD-12	Long	Yellow	21.49 ± 2.44	2.29	Hubei, China
JYD-13	Oval	Yellow	15.12 ± 2.03	1.25	Hubei, China
JYD-14	Long	Yellow	18.8 ± 0.97	2.09	Hubei, China
JYD-15	Long	Yellow	17.52 ± 1.36	1.54	Henan, China
JYD-16	Round	Yellow	14.93 ± 1.56	1.17	Henan, China
JYD-17	Oval	Yellow	15.52 ± 1.9	1.21	Henan, China
JYD-18	Oval	Yellow	16.6 ± 0.98	1.28	Henan, China
JYD-19	Oval	Yellow	15.17 ± 0.59	1.28	Henan, China
JYD-20	Long	Yellow	18.46 ± 2.33	2.04	Henan, China
JYD-21	Round	Yellow	15.59 ± 0.7	1.18	Jiangsu, China
JYD-22	Oval	Yellow	14.79 ± 0.56	1.29	Jiangsu, China
JYD-23	Round	Yellow	14.98 ± 1.16	1.12	Jilin, China
JYD-24	Round	Yellow	13.26 ± 1.23	1.12	Jilin, China
JYD-25	Oval	Yellow	13.97 ± 1.09	1.20	Jilin, China
JYD-26	Round	Yellow	13.78 ± 0.68	1.15	Hebei, China
JYD-27	Long	Yellow	17.17 ± 1.11	2.16	Hebei, China
JYD-28	Round	Yellow	13.13 ± 0.5	1.15	Hebei, China
JYD-29	Long	Yellow	19.45 ± 2.47	2.06	Jilin, China
JYD-30	Round	Yellow	15.58 ± 1.91	1.19	Jilin, China
JYD-31	Oval	Yellow	15.01 ± 0.67	1.23	Hubei, China
JYD-32	Oval	Yellow	13.91 ± 0.51	1.25	Ghana
JYD-33	Oval	Yellow	16.07 ± 2.43	1.40	Mali
JYD-34	Oval	Red	16.22 ± 1.64	1.34	Cameron
JYD-35	Oval	Yellow	9.52 ± 1.64	1.30	Cameron
JYD-36	Oval	Yellow	14.17 ± 0.68	1.23	Cameron
JYD-37	Round	Yellow	14.15 ± 0.8	1.13	Jilin, China
JYD-38	Round	Yellow	15.23 ± 0.83	1.08	Jilin, China
JYD-39	Oval	Yellow	15.35 ± 0.46	1.31	Hebei, China
JYD-40	Long	Yellow	19.94 ± 2.56	2.18	Hebei, China
JYD-41	Long	Yellow	25.3 ± 2.76	1.62	Russia
JYD-42	Oval	Yellow	14.06 ± 1.47	1.29	Spain

Note: Shape of the tuber was classified based on the length/width ratio of the fresh mature tubers: round (1.0–1.2), oval (1.2–1.5), and long (>1.5). TLWR—tuber length/width ratio.

**Table 2:** Descriptive statistics of agronomic and biochemical traits under field conditions.

Agronomic Traits	Year	Descriptive Statistics			
		Min. Value	Max. Value	Mean ± SD	CV(%)
PH (cm)	2019	75.30	149.15	122.99 ± 13.98	11.37
	2020	92.04	168.95	140.96 ± 15.01	10.65
LW (mm)	2019	3.70	8.35	5.49 ± 0.97	17.67
	2020	4.08	9.05	6.3 ± 1.13	17.94
LLWR	2019	15.86	35.22	22.98 ± 4.39	19.10
	2020	15.88	35.03	22.96 ± 4.38	19.08
TL (mm)	2019	8.85	22.77	15.01 ± 2.43	16.19
	2020	10.19	27.83	16.85 ± 2.8	16.62
TW (mm)	2019	7.26	15.50	11.19 ± 1.76	15.73
	2020	7.36	15.82	11.45 ± 1.79	15.63
TLWR	2019	1.05	2.21	1.38 ± 0.37	26.81
	2020	1.11	2.37	1.51 ± 0.39	25.83
TN	2019	15.77	81.78	31.84 ± 15.92	50.00
	2020	18.02	92.22	36.18 ± 18.39	50.83
100 TW(g)	2019	33.84	221.84	99.28 ± 29.54	29.75
	2020	34.32	236.04	102.85 ± 31.24	30.37
GY (kg/667 m^2^)	2019	385.71	1212.68	747.06 ± 191.07	25.58
	2020	394.29	1227.32	785.02 ± 190.92	24.32
TY (kg/667 m^2^)	2019	619.92	1325.7	947.38 ± 194.44	20.52
	2020	765.38	1514.95	1110.67 ± 252.02	22.69
Starch content (%)	2020	17.92	27.20	21.62 ± 2.07	9.57
Oil content (%)	2020	14.04	21.28	17.97 ± 1.61	8.96
Sugars content (%)	2020	17.23	25.56	20.37 ± 2.04	10.01
Protein content (%)	2020	4.93	7.90	6.02 ± 0.65	10.80

Note: PH—plant height; LW—leaf width; LLWR—leaf length/width ratio; TL—tuber length; TW—tuber width; TLWR—tuber length/width ratio; TN—tiller number; 100 TW—100-tuber weight; GY—grass yield per 667 m^2^; TY—tuber yield per 667 m^2^.

**Table 3:** ANOVA analysis of the agronomic and biochemical traits in three tiger nut groups with different tuber shapes.

Traits	Group I (Round)	Group II (Oval)	Group III (Long)
PH (cm)	137.87 ± 12.24	128.91 ± 16.92	128.88 ± 10.17
LW (mm)	6.54 ± 1.18	6.08 ± 0.81	5.13 ± 0.81
LLWR	21.75 ± 4.67	21.45 ± 3.51	25.64 ± 4.30
TN	23.63 ± 6.40	28.44 ± 12.13	53 ± 18.40
TL (mm)	14.49 ± 0.91	14.74 ± 1.52	19.12 ± 2.46
TW (mm)	12.61 ± 0.83	11.61 ± 1.21	9.79 ± 2.14
TLWR	1.15 ± 0.04	1.27 ± 0.05	1.99 ± 0.24
100 TW(g)	103.5 ± 26.83	101.12 ± 24.73	100.38 ± 44.96
GY (kg/667 m^2^)	675.45 ± 167.43	701.15 ± 153.17	938.5 ± 175.85
TY (kg/667 m^2^)	1028.57 ± 207.46	930.08 ± 197.9	1203.3 ± 158.31
Protein content (%)	5.74 ± 0.30	5.83 ± 0.65	6.61 ± 0.61
Oil content (%)	18.01 ± 1.83	18.28 ± 1.40	16.96 ± 1.41
Starch content (%)	21.71 ± 1.60	21.89 ± 2.35	20.95 ± 1.48
Sugar content (%)	21.03 ± 2.54	20.36 ± 1.83	19.79 ± 1.99

Note: PH—plant height; LW—leaf width; LLWR—leaf length/width ratio; TL—tuber length; TW—tuber width; TLWR—tuber length/width ratio; TN—tiller number; 100 TW—100-tuber weight; GY—grass yield per 667 m^2^; TY—tuber yield per 667 m^2^.

**Table 4:** Correlation coefficients of 14 agronomic and biochemical traits among 42 accessions.

Traits	PH	LW	LLWR	TN	TL	TW	TLWR	100 TW	GY	TY	Protein	Oil	Starch
LW	0.20												
LLWR	0.43 **	−0.78 **											
TN	−0.24	−0.41 **	0.27										
TL	0.07	−0.27	0.31 *	0.49 **									
TW	0.42 **	0.60 **	−0.33 *	−0.56 **	−0.06								
TLWR	−0.21	−0.61 **	0.47 **	0.71 **	0.71 **	−0.73 **							
100 TW	0.27	0.44 **	−0.24	−0.05	0.33 *	0.60 **	−0.21						
GY	0.11	−0.29	0.37 *	0.57 **	0.56 **	−0.21	0.53 **	0.13					
TY	−0.18	−0.12	0.06	0.40 **	0.46 **	−0.23	0.49 **	0.07	0.26				
Protein	−0.32 *	−0.44 **	0.22	0.72 **	0.18	−0.66 **	0.56 **	−0.21	0.17	0.28			
Oil	0.32 *	0.22	−0.1	−0.43 **	−0.15	0.40 **	−0.39 *	0.21	−0.19	−0.24	−0.39 *		
Starch	0.11	0.14	−0.10	−0.15	0.02	0.36 *	−0.24	0.32 *	0.11	−0.01	−0.31 *	0.39 *	
Sugar	0.24	0.19	−0.02	−0.23	−0.23	−0.04	−0.11	−0.32 *	−0.11	−0.09	−0.14	0.07	−0.38 *

* and ** denote the significant differences at *p* < 0.05 and *p* < 0.01, respectively. Note: PH—plant height; LW—leaf width; LLWR—leaf length/width ratio; TL—tuber length; TW—tuber width; TLWR—tuber length/width ratio; TN—tiller number; 100 TW—100-tuber weight; GY—grass yield per 667 m^2^; TY—tuber yield per 667 m^2^.

**Table 5:** First 4 principal components based on 14 traits by PCA analysis.

Traits	PV1	PV2	PV3	PV4
PH	−0.31	0.39	0.70	0.28
LW	−0.73	0.11	−0.40	0.43
LLWR	0.53	0.15	0.77	−0.16
TN	0.82	0.20	−0.21	0.04
TL	0.53	0.68	−0.01	0.22
TW	−0.80	0.45	0.00	0.11
TLWR	0.92	0.15	0.03	0.09
100 TW	−0.33	0.76	−0.27	0.14
GY	0.54	0.53	0.15	0.12
TY	0.48	0.26	−0.32	0.34
Protein	0.74	−0.19	−0.22	−0.06
Oil	−0.54	0.21	0.28	−0.21
Starch	−0.33	0.52	−0.06	−0.57
Suger	−0.16	−0.44	0.38	0.65
Eigenvalue	4.93	2.38	1.76	1.30
Contribution rate CR(%)	35.18	17.01	12.60	9.27
Cumulative contribution rate CCR(%)	35.18	52.16	64.80	74.07

Note: PH—plant height; LW—leaf width; LLWR—leaf length/width ratio; TL—tuber length; TW—tuber width; TLWR—tuber length/width ratio; TN—tiller number; 100 TW—100-tuber weight; GY—grass yield per 667 m^2^; TY—tuber yield per 667 m^2^.

**Table 6:** Comprehensive assessment of 42 tiger nut accessions based on the 14 agronomic and biochemical traits.

Accessions	F-Value	Rank	Accessions	F-Value	Rank	Accessions	F-Value	Rank
JYD-1	3.8	12	JYD-15	3.34	19	JYD-29	4.01	4
JYD-2	3.09	24	JYD-16	3.58	16	JYD-30	3.88	9
JYD-3	3.66	14	JYD-17	2.51	39	JYD-31	2.66	34
JYD-4	2.88	30	JYD-18	3.42	18	JYD-32	2.61	37
JYD-5	3.92	7	JYD-19	2.98	28	JYD-33	2.75	33
JYD-6	3.07	25	JYD-20	4.3	3	JYD-34	3.81	11
JYD-7	3.92	8	JYD-21	3.28	21	JYD-35	2.78	32
JYD-8	3.01	26	JYD-22	2.91	29	JYD-36	2.59	38
JYD-9	3.0	27	JYD-23	3.28	22	JYD-37	2.38	42
JYD-10	3.1	23	JYD-24	2.85	31	JYD-38	2.64	36
JYD-11	3.29	20	JYD-25	3.86	10	JYD-39	2.66	35
JYD-12	3.93	6	JYD-26	3.55	17	JYD-40	3.64	15
JYD-13	3.71	13	JYD-27	4.72	1	JYD-41	4.33	2
JYD-14	3.98	5	JYD-28	2.46	41	JYD-42	2.51	40

**Table 7:** Correlation coefficients between 14 phenotypic traits and comprehensive value.

Trait	F-Value	Trait	F-Value
PH	−0.073	100 TW	0.09
LW	−0.294	GY	0.717 **
LLWR	0.292	TY	0.850 **
TN	0.669 **	Protein	0.381 *
TL	0.653 **	Oil	−0.307 **
TW	−0.336 *	Starch	−0.068
TLWR	0.690 **	Suger	−0.136

Note: PH—plant height; LW—leaf width; LLWR—leaf length/width ratio; TL—tuber length; TW—tuber width; TLWR—tuber length/width ratio; TN—tiller number; 100 TW—100-tuber weight; GY—grass yield per 667 m^2^; TY—tuber yield per 667 m^2^, * and ** denote the significant differences at *p* < 0.05 and *p* < 0.01, respectively..
